# The Impact of Nutrition and Fine Particulate Matter (PM2.5) on Inflammatory Responses in Individuals with Metabolic Syndrome: A Paired Case Study from Chiang Mai, Thailand

**DOI:** 10.3390/toxics13050325

**Published:** 2025-04-22

**Authors:** Wason Parklak, Hataichanok Chuljerm, Sawaeng Kawichai, Puriwat Fakfum, Putita Jiraya, Praporn Kijkuokool, Wiritphon Khiaolaongam, Pakaphorn Ngamsang, Sakaewan Ounjaijean, Kittipan Rerkasem, Kanokwan Kulprachakarn

**Affiliations:** 1 Research Center for Non-Infectious Diseases and Environmental Health, Research Institute for Health Sciences, Chiang Mai University, Chiang Mai 50200, Thailand; wason.p@cmu.ac.th (W.P.); hataichanok.ch@cmu.ac.th (H.C.); sawaeng.kaw@cmu.ac.th (S.K.); nuengpuriwat@gmail.com (P.F.); putita_jiraya@cmu.ac.th (P.J.); sakaewan.o@cmu.ac.th (S.O.); rerkase@gmail.com (K.R.); 2 School of Health Sciences Research, Research Institute for Health Sciences, Chiang Mai University, Chiang Mai 50200, Thailand; praporn_k@cmu.ac.th (P.K.); wiritphon_k@cmu.ac.th (W.K.); pakaporn_ng@cmu.ac.th (P.N.); 3 Clinical Surgical Research Center, Department of Surgery, Faculty of Medicine, Chiang Mai University, Chiang Mai 50200, Thailand

**Keywords:** PM2.5, nutrition, inflammation, dietary fiber, antioxidant vitamins, metabolic syndrome

## Abstract

Exposure to fine particulate matter (PM2.5) is linked to increased systemic inflammation, particularly in individuals with metabolic syndrome (MS). This study assessed the impact of nutrition and PM2.5 exposure on inflammatory markers in individuals with MS. A total of 50 participants (25 with MS, 25 healthy controls) were monitored during a high-PM2.5 exposure period (HEP) and a low-PM2.5 exposure period (LEP). Dietary intake, health assessments, and inflammatory markers—TNF-α, IL-6, and CRP—were evaluated. The MS group had significantly higher BMI, fasting blood glucose, and triglyceride levels and lower HDL-C than the healthy group (*p* < 0.01), but these parameters did not change significantly between the HEP and LEP. Notably, dietary fiber intake increased in the MS group during the LEP (*p* < 0.05). CRP levels were higher in the MS group and significantly decreased in both groups during the LEP (*p* < 0.05). IL-6 was higher in the MS group during the HEP but did not significantly change across periods. TNF-α showed no differences. Dietary fiber intake was inversely correlated with IL-6 and CRP in the healthy group and strongly correlated with CRP in the MS group (*r* = −0.403, *p* < 0.01). Antioxidant vitamins were inversely correlated with inflammation only in healthy participants. These findings suggest that an increased dietary fiber intake may help reduce PM2.5-induced inflammation, particularly in individuals with MS.

## 1. Introduction

Fine particulate matter (PM2.5), airborne particles smaller than 2.5 μm in diameter, poses significant health risks due to its ability to penetrate deep into the respiratory tract and enter the bloodstream [1]. Exposure to PM2.5 is associated with respiratory diseases and cardiovascular diseases (CVDs) and has recently been linked to systemic inflammation, a key driver of chronic conditions [2,3,4]. PM2.5 induces inflammation through oxidative stress and the activation of inflammatory pathways [4]. Once inhaled, PM2.5 generates reactive oxygen species (ROS), leading to oxidative damage and the activation of nuclear factor-kappa B (NF-κB), which triggers the release of pro-inflammatory cytokines such as tumor necrosis factor-alpha (TNF-α) and interleukin-6 (IL-6) [5,6]. These cytokines stimulate the liver to produce acute-phase proteins like C-reactive protein (CRP), a systemic inflammatory marker [7].

Individuals with metabolic syndrome (MS), characterized by central obesity, hypertension, insulin resistance, and dyslipidemia [8], are particularly vulnerable to PM2.5 exposure [9,10]. Prolonged exposure to air pollution is associated with an increased risk of MS, suggesting that PM2.5 exacerbates metabolic dysfunction through inflammatory processes [11]. Experimental studies in animal models have demonstrated that PM2.5 exposure induces a wide range of metabolic impairments, including glucose intolerance, insulin resistance, dyslipidemia, and disturbances in energy metabolism. These metabolic alterations are closely associated with enhanced inflammatory responses in the respiratory tract, circulation, and visceral adipose tissue (VAT), characterized by elevated levels of pro-inflammatory cytokines such as IL-6 and TNF-α in the lungs, serum, and VAT [12]. Additionally, Zhang et al. (2023) reported that PM2.5 exposure leads to a reduction in energy expenditure, including decreased oxygen consumption, heat production, and respiratory exchange ratio. These changes contribute to the development of hepatic steatosis (fatty liver) and dyslipidemia, which are hallmarks of metabolic dysfunction [13].

At the molecular level, PM2.5 alters the expression of key genes involved in lipid metabolism. Specifically, it downregulates the expression of peroxisome proliferator-activated receptors alpha and gamma (PPARα and PPARγ), which play essential roles in fatty acid oxidation and lipid regulation, while upregulating sterol regulatory element-binding protein 1 (SREBP1), a transcription factor that promotes lipogenesis. These gene expression changes exacerbate hepatic lipid accumulation and contribute to liver metabolic disorders [13]. Moreover, emerging evidence suggests that even short-term exposure to PM2.5 can induce hypothalamic inflammation, mimicking the physiological effects of a high-fat diet. This neuro-inflammatory response may impair the central regulation of energy balance and metabolism, thereby worsening metabolic syndrome-related dysfunction [14].

PM2.5 exposure has been shown to increase oxidative stress and chronic low-grade inflammation, both of which contribute to MS progression. However, dietary interventions can help mitigate these effects [15,16,17]. Antioxidant nutrients, including vitamins A, C, and E, as well as essential trace minerals like zinc (Zn) and copper (Cu), have been demonstrated to counteract oxidative stress and inflammatory pathways [17,18,19]. Vitamin C, a potent water-soluble antioxidant, scavenges ROS and enhances endothelial function, reducing vascular inflammation [20]. Vitamin E, a lipid-soluble antioxidant, protects cellular membranes from oxidative damage and is linked to reduced levels of inflammatory markers such as CRP and IL-6 [21,22]. Similarly, vitamin A and its active metabolites, retinoids, regulate immune responses and suppress pro-inflammatory cytokines, reducing systemic inflammation [23]. Zn and Cu play crucial roles in antioxidant enzyme systems, such as superoxide dismutase, which neutralizes oxidative stress and supports immune function [24]. Evidence from epidemiological and interventional studies suggests that diets rich in antioxidants and polyphenols significantly reduce inflammation and oxidative stress, improving metabolic health, in individuals with MS [25,26]. Given the strong connection between diet, inflammation, and metabolic dysfunction, adopting an antioxidant-rich diet may serve as an effective strategy to manage MS and reduce its complications.

Northern Thailand, particularly Chiang Mai Province, experiences severe air pollution caused by PM2.5 annually, with pollution peaking between January and April due to agricultural burning and forest fires [27,28]. The region’s mountainous terrain creates basin-like areas that trap air pollutants, exacerbating pollution levels. Samoeng District, in particular, is a high-risk area due to its valley-like geography, which allows smoke from forest fires to accumulate, leading to hazardous air quality. In April 2019, the Air Quality Index (AQI) in Samoeng District exceeded 500—the maximum measurable level—indicating extreme pollution [28,29]. This persistent air pollution significantly impacts public health, increasing the risk of respiratory diseases and CVDs. PM2.5 particles can penetrate deep into the lungs and enter the bloodstream, triggering widespread inflammation and health complications [1].

Given these environmental and health concerns, this study aims to evaluate the impact of nutrition and PM2.5 exposure on inflammatory responses in individuals with metabolic syndrome in Chiang Mai, Thailand. Understanding the role of dietary factors in mitigating PM2.5-induced inflammation may provide valuable insights for developing public health strategies in affected regions.

## 2. Materials and Methods

### 2.1. Air Quality Monitoring and PM2.5 Measurement

**Figure 1:**
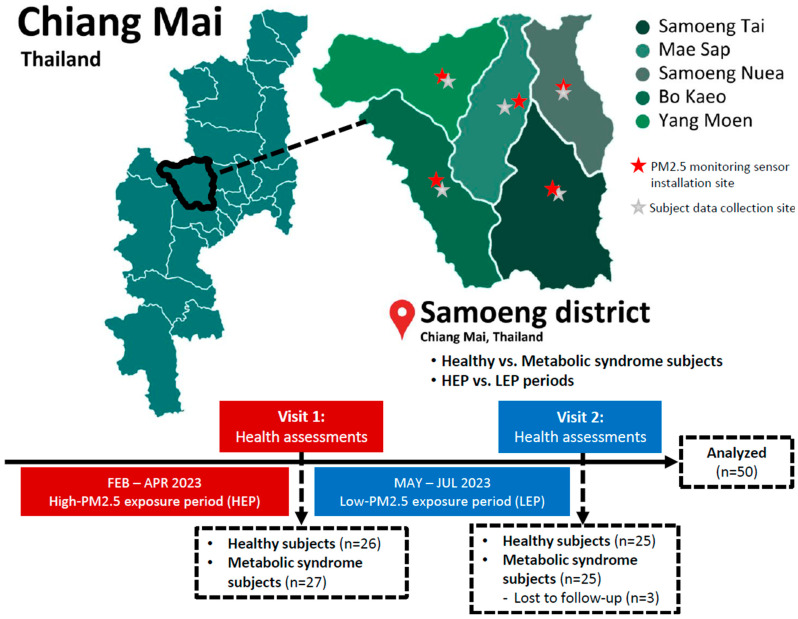
Flowchart of study design for data collection and paired comparisons between healthy and metabolic syndrome groups, and between high- and low-PM2.5 exposure periods.

Air quality sensors were installed in the areas of the Subdistrict Administrative Organizations in each subdistrict of Samoeng District, Chiang Mai Province. These sensors continuously monitored ambient air quality and PM2.5 concentrations. The focus was on five subdistricts: Samoeng Tai, Samoeng Nuea, Mae Saab, Bo Kaeo, and Yang Moen (sensor installation locations are marked with red stars in Figure 1). Model PMS7003 of the PM2.5 sensor was manufactured by Beijing Plantower Co., Ltd. in Beijing, China, and was assembled as a final product with a relative humidity correction of PM2.5 by Nanogeneration Co., Ltd., in Chiang Mai, Thailand. After a laser beam passes through the air sample, the PMS7003 photodiode detector is used to measure the intensity of scattered light. This method is employed to determine the number of particles per unit volume and the equivalent grain size of particles based on varying particle diameters. The levels of PM2.5 were measured and recorded daily during two distinct exposure periods: February–April 2023 (the high-PM2.5 exposure period) and May–July 2023 (the low-PM2.5 exposure period). The air quality data were obtained from the Northern Thailand Air Quality Health Index (NTAQHI) system (https://www2.ntaqhi.info/, accessed on 21 April 2025), which is administered by the Research Institute for Health Sciences (RIHES), Chiang Mai University. PM2.5 measurements were obtained by employing sensors that were developed by the Environmental and Occupational Health Unit at RIHES. Each data averaging method and quality control phase was implemented in accordance with the established protocols of RIHES. Thus, the measurements were guaranteed to be precise and dependable.

### 2.2. Ethics Statement

This study complied with the principles of the Declaration of Helsinki, and all procedures involving human participants were approved by the Human Experimentation Committee at the Research Institute for Health Sciences, Chiang Mai University. Ethical approval was granted on 19 January 2023 (approval number: 03/2023). Prior to participation, all subjects provided written informed consent.

### 2.3. Study Subjects

This study included both healthy individuals and individuals with metabolic syndrome (MS) from Samoeng District, Chiang Mai Province, Thailand. Participants were equally selected from five subdistricts of Samoeng District: Samoeng Tai, Samoeng Nuea, Mae Saab, Bo Kaeo, and Yang Moen. The selection and data collection were conducted at Subdistrict Health Promoting Hospitals and the District Hospital (subject screening and data collection sites are marked with gray stars in Figure 1). The selection of these areas was based on their relevance to the study objectives, particularly in assessing environmental exposure. Participants were required to be male or female, aged between 25 and 60 years. For the healthy group, individuals were eligible if they had no history of serious diseases such as cancer, CVDs, or chronic kidney disease and did not have any active infections at the time of recruitment. In contrast, individuals in the MS group were required to meet at least three of the established diagnostic criteria for MS. These criteria included a fasting blood glucose (FBG) level of at least 100 mg/dL after an eight-hour fasting period, a waist circumference (WC) exceeding 90 cm for males and 80 cm for females, elevated blood pressure of 130/85 mmHg or higher, triglyceride (TG) levels of at least 150 mg/dL, and reduced high-density lipoprotein cholesterol (HDL-C) levels, defined as less than 40 mg/dL for males and less than 50 mg/dL for females. To ensure consistency in the environmental exposure assessment, participants in both groups were required to have resided in Samoeng District for a minimum of five years.

Individuals were excluded from the study if they failed to provide full cooperation, such as missing scheduled visits or losing contact with the research team. Additional exclusion criteria included having undergone recent surgery, being pregnant, a history of substance abuse, or the presence of neurological or psychiatric disorders. Furthermore, individuals with active infections at the time of recruitment were excluded to minimize confounding effects on the study outcomes.

### 2.4. Study Design

This study is a prospective observational study designed to assess the impact of PM2.5 exposure on health outcomes. A paired comparison was conducted across two time periods: the high-PM2.5 exposure period (HEP) and the low-PM2.5 exposure period (LEP). Additionally, a paired comparison was performed to evaluate differences in health effects between healthy subjects and those with MS. The study design and comparative framework are illustrated in Figure 1. Following ethical approval from the Human Experimentation Committee at the Research Institute for Health Sciences, Chiang Mai University, the research team coordinated with local health authorities in each subdistrict of Samoeng District to conduct preliminary health screenings for participant recruitment. A total of 53 participants were enrolled in the study, comprising 26 healthy individuals and 27 individuals with MS, recruited proportionally from various subdistricts within Samoeng District.

Following the HEP (February–April 2023, Visit 1), data collection was conducted in late April 2023 using structured questionnaires to obtain demographic information and dietary intake data. Blood samples were collected from all participants between 7:00 and 9:00 a.m., following an overnight fast of at least 10–12 h. Venipuncture was performed by trained medical personnel. All samples were processed within 1 h of collection. Serum and plasma were separated by centrifugation at 3000 rpm for 10 min at 4 °C, and then stored at −80 °C until further analysis of metabolic parameters and inflammatory markers. Additionally, anthropometric measurements, including body weight, height, waist circumference, hip circumference, and blood pressure, were recorded.

Following the LEP (May–July 2023, Visit 2), in late July 2023, participants underwent a second round of data collection, following the same protocol as in Visit 1, to assess changes in metabolic and inflammatory responses in relation to PM2.5 exposure. However, during Visit 2, 3 participants were lost to follow-up, leaving a total of 50 participants (25 healthy individuals and 25 individuals with MS) for final analysis.

### 2.5. Laboratory Procedures

Blood samples collected from participants during both the HEP and LEP were analyzed to assess biochemical profiles and inflammatory markers. An automated biochemistry analyzer (Rx Daytona, Randox Laboratories Ltd., Antrim, UK) was employed to analyze biochemical profiles, which included fasting blood glucose, lipid profile, and liver and renal function parameters. Quality control procedures were implemented during installation and routine operation to guarantee data accuracy and reliability. All reagent batches were calibrated, and the within-run precision was evaluated using 20 replicates per test. For lipid parameters and fasting blood glucose, the acceptable %CV thresholds were established at ≤5%, while those for liver and renal markers were set at ≤10%. Commercial control sera at various concentrations were administered prior to each session during routine testing, and calibration was repeated as required. The validity and reproducibility of the biochemical data were guaranteed by only proceeding with the tests when all quality control results met acceptance criteria.

Spectrophotometric measurements of inflammatory markers, including TNF-α, IL-6, and CRP, were performed with the Human Tumor Necrosis Factor-α ELISA Kit (cat. no. RAB0476-1KT), the Human IL-6 ELISA Kit (cat. no. RAB0306-1KT), and the Human C-Reactive Protein ELISA Kit (cat. no. RAB0096-1KT), respectively. All ELISA kits were obtained from Sigma Aldrich (St. Louis, MO, USA), and the assays were performed according to the manufacturer’s protocols to ensure data accuracy and reliability. To make sure the analysis was accurate, all measurements were performed in duplicate, and standard curves were created for each test using a 4-parameter logistic fit. Only assays with a coefficient of determination R^2^ > 0.990 were accepted. All samples for each marker were tested on the same day to reduce differences between tests and to avoid repeated freezing and thawing, which could harm protein molecules and affect the accuracy of the tests. The absorbance of each assay was measured using a BMG SPECTROstar Nano2 microplate reader (BMG Labtech, Ortenberg, Germany).

### 2.6. Dietary Intake Data

Dietary intake data were collected using a 24 h dietary recall questionnaire, conducted over three non-consecutive days, including two weekdays and one weekend day. Energy intake and nutrient intake were analyzed using INMUCAL-Nutrients version 4.0, a software developed by the Institute of Nutrition, Mahidol University, Thailand. The results were reported as the average daily intake for each participant.

### 2.7. Statistical Analysis

All statistical analyses were performed using SPSS software, version 15.0 (SPSS Inc., Chicago, IL, USA). Descriptive statistics were used to summarize baseline characteristics. Continuous variables are presented as mean ± standard deviation (SD), while categorical variables are expressed as counts (*n*) and percentages (%). Categorical variables, including the demographic characteristics, lifestyle factors, and health conditions of participants, such as sex, age group, smoking status, alcohol consumption, and the presence of chronic diseases, were considered potential confounding factors that may influence both dietary intake and inflammatory marker levels. These variables were compared between groups using Chi-square tests. The unpaired *t*-test was used to compare continuous variables between independent groups, while the paired *t*-test was used to compare continuous variables between the two exposure periods (HEP vs. LEP) within the same subjects. Both tests had a *p*-value of <0.05. The association between nutrient intake and inflammatory markers was analyzed using data from both the HEP and LEP (*n* = 50). The analysis was conducted separately for each group of participants. Pearson correlation coefficients (*r*) were calculated to assess relationships, and statistical significance was determined using a two-tailed test with a *p*-value of <0.05.

### 2.8. Artificial Intelligence (AI) for Research

During the preparation of this study/manuscript, the authors used AI tools including ChatGPT (GPT-4o) to assist in retrieving relevant scientific information. The authors thoroughly reviewed and verified the accuracy of the information before incorporating it into the study and manuscript writing. Additionally, QuillBot (v19.31.0) was used for language refinement and grammatical corrections. The core content and key ideas remain entirely the authors’ original work.

## 3. Results

### 3.1. PM2.5 Levels and Air Quality Health Index (AQHI) in Samoeng District

**Figure 2:**
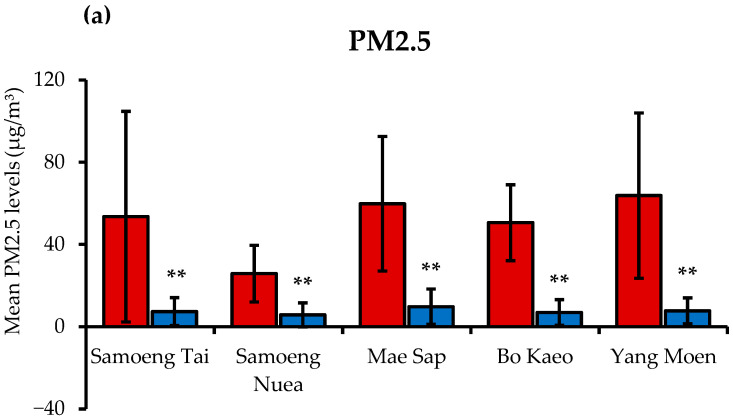

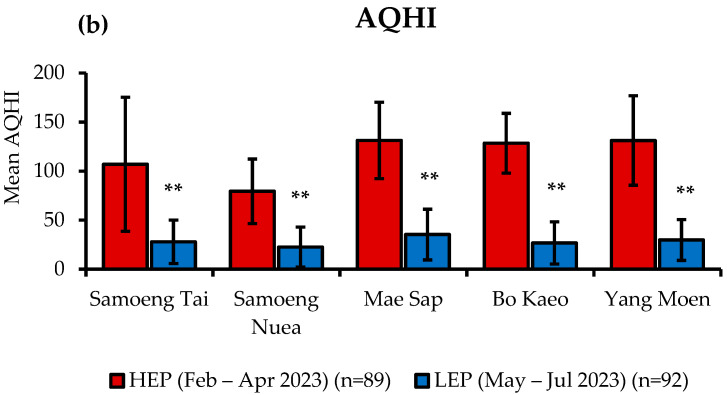
The PM2.5 levels (**a**) and Air Quality Health Index (AQHI) (**b**) in five subdistricts of Samoeng District, including Samoeng Tai, Samoeng Nuea, Mae Sap, Bo Kaeo, and Yang Moen. Data are presented as mean ± standard deviation (SD) for the high-level PM2.5 exposure period (HEP) from February to April 2023 (*n* = 89) and the low-level PM2.5 exposure period (LEP) from May to July 2023 (*n* = 92). ** indicates a statistically significant difference within the same subdistrict between the two exposure periods with a *p*-value of <0.01.

The analysis of PM2.5 levels and the Air Quality Health Index (AQHI) in various subdistricts of Samoeng District was conducted using data from the Northern Thailand Air Quality Health Index (NTAQHI). The mean values were calculated for both the HEP (February–April 2023) and the LEP (May–July 2023). As illustrated in Figure 2a, the findings indicate that the mean PM2.5 levels significantly decreased from the HEP to the LEP across all subdistricts (*p* < 0.01). The highest mean PM2.5 concentration during the HEP was recorded in Yang Moen (63.76 ± 40.20 µg/m^3^), whereas the lowest mean PM2.5 concentration was observed in Samoeng Nuea (25.80 ± 13.74 µg/m^3^). Conversely, during the LEP, PM2.5 levels substantially declined, with Bo Kaeo recording the lowest mean PM2.5 concentration (6.92 ± 6.23 µg/m^3^). Similarly, the mean AQHI values (as shown in Figure 2b) were significantly higher during the HEP compared to the LEP across all subdistricts (*p* < 0.01). The highest AQHI value was observed in Yang Moen (131.16 ± 45.57), while Samoeng Nuea recorded the lowest AQHI value (79.49 ± 32.92). During the LEP, the AQHI values decreased, with the lowest mean AQHI value recorded in Samoeng Nuea (22.54 ± 20.44).

When averaging data across all five subdistricts, the HEP exhibited significantly higher mean PM2.5 concentrations and AQHI values compared to the LEP (*p* < 0.001 for both). Specifically, the mean PM2.5 concentration during the HEP was 50.86 ± 37.98 µg/m^3^, while that during the LEP was 7.48 ± 6.91 µg/m^3^. Correspondingly, the mean AQHI values were 114.30 ± 50.93 during the HEP and 28.40 ± 22.53 during the LEP.

### 3.2. The Demographic Data of Subjects

Table 1 presents the findings on subject characteristics and health conditions. This study compared demographic factors, lifestyle behaviors, and underlying health conditions between healthy individuals (*n* = 25) and individuals with MS (*n* = 25), with a total sample size of 50 subjects. The results indicate no statistically significant difference in gender distribution between the two groups (*p* = 0.225), although a higher proportion of females was observed in both the healthy group (60.0%) and the MS group (76.0%). The mean age was slightly higher in individuals with MS (52.2 ± 8.3 years) compared to healthy individuals (48.3 ± 14.3 years), but this difference was not statistically significant (*p* = 0.177). Regarding lifestyle behaviors, alcohol consumption was more frequent among healthy individuals (76.0%) than those with MS (52.0%), although this difference did not reach statistical significance (*p* = 0.140). Similarly, there was no significant difference in smoking status between the two groups (*p* = 0.411), with the majority of participants being non-smokers (76.0%), followed by smokers (10.0%) and former smokers (14.0%).

Additionally, diabetes was observed only in the metabolic syndrome group (10.0%), but this difference did not reach statistical significance (*p* = 0.050). However, hypertension and dyslipidemia were significantly more prevalent in individuals with MS. Hypertension was significantly higher in the MS group (56.0%) compared to the healthy group (8.0%) (*p* = 0.001). Similarly, dyslipidemia was significantly more common in the MS group (28.0%) than in the healthy group (4.0%) (*p* = 0.049).

### 3.3. Effects of PM2.5 Exposure on Physical and Blood Biochemical Parameters

Table 2 displays the physical and biochemical data of 25 healthy individuals and 25 individuals with MS following the HEP and LEP. Body weight and body mass index (BMI) were significantly higher in individuals with MS compared to healthy subjects in both the HEP (*p* < 0.01) and LEP (*p* < 0.01). Waist circumference (WC) and hip circumference (HC) were also significantly larger in the MS group compared to the healthy group in both exposure periods (*p* < 0.01, *p* < 0.01). Systolic blood pressure (SBP) and diastolic blood pressure (DBP) did not significantly differ between the healthy and MS groups after either exposure period. However, in the healthy group, both SBP and DBP tended to decrease during the LEP compared to the HEP, with SBP decreasing from 134.6 ± 19.8 mmHg to 125.6 ± 19.6 mmHg and DBP decreasing from 83.8 ± 10.6 mmHg to 78.2 ± 10.2 mmHg, though these differences were not statistically significant (SBP, *p* = 0.056; DBP, *p* = 0.057). Heart rate (HR) did not differ significantly between the healthy and MS groups after either exposure period.

For blood biochemical parameters, fasting blood glucose (FBG) was significantly higher in the MS group compared to the healthy group in both the HEP (*p* < 0.01) and LEP (*p* < 0.01). Total cholesterol (TC) levels did not differ significantly between groups in either exposure period. Triglyceride (TG) levels were significantly higher in individuals with MS compared to healthy subjects in both the HEP (*p* < 0.01) and LEP (*p* < 0.01). High-density lipoprotein cholesterol (HDL-C) levels were significantly lower in the MS group than in the healthy group (*p* = 0.001, *p* = 0.034). However, low-density lipoprotein cholesterol (LDL-C) levels did not show significant differences.

Liver function parameters, including alanine aminotransferase (ALT) and aspartate aminotransferase (AST), did not differ significantly between groups, except for ALT, which was significantly higher in the MS group during the LEP (*p* = 0.040). Serum creatinine levels were significantly higher in the MS group during the LEP (*p* = 0.047). Blood urea nitrogen (BUN) levels did not differ significantly between groups or across exposure periods.

### 3.4. Energy and Nutrient Intakes

#### 3.4.1. Distribution of Energy Percentages from Macronutrients

The distribution of energy intake from macronutrients in healthy subjects and individuals with MS during the HEP and LEP is presented in Table 3. The results indicate that the percentage of energy intake from carbohydrates, proteins, and fats did not differ significantly between the healthy and MS groups in either the HEP or LEP. During the HEP, carbohydrate intake contributed to 56.96 ± 9.23% of total energy in the healthy group and 54.24 ± 17.34% in the MS group (*p* = 0.493), while during the LEP, carbohydrate intake represented 53.48 ± 16.82% of total energy in the healthy group and 53.25 ± 13.94% in the MS group (*p* = 0.960). Similarly, the proportion of protein intake did not show significant differences between the groups. In the HEP, proteins accounted for 17.44 ± 5.13% of total energy in the healthy group and 19.20 ± 11.62% in the MS group (*p* = 0.491), while during the LEP, proteins contributed to 18.69 ± 10.6% of total energy in the healthy group and 19.68 ± 7.57% in the MS group (*p* = 0.613). The percentage of fat intake was also comparable between groups, with 25.60 ± 8.26% of total energy derived from fats in the healthy group and 26.55 ± 11.70% in the MS group during the HEP (*p* = 0.741). In the LEP, the fat intake was 27.83 ± 12.74% in the healthy group and 27.06 ± 9.23% in the MS group (*p* = 0.808).

#### 3.4.2. Average Daily Energy and Nutrient Consumption

Table 4 presents the average daily energy and food consumption of healthy subjects and those with MS during the HEP and LEP. The results show that total energy intake, macronutrient intake (carbohydrates, proteins, and fats), and total saturated fatty acid intake did not significantly differ between the healthy and MS groups in either the HEP or the LEP. Similarly, no significant differences were observed in the intake of cholesterol, calcium, phosphorus, iron, potassium, sodium, magnesium, copper, selenium, and zinc between the two groups during either exposure period. For vitamin intake, no significant differences were found in vitamins B1, B2, B6, B12, and niacin between the healthy and MS groups in both the HEP and LEP. When comparing dietary intake between the HEP and LEP within the same group, a statistically significant decrease in dietary fiber intake was observed in the MS group during the LEP compared to the HEP (*p* = 0.037). Additionally, during the HEP, the healthy group exhibited a trend toward a higher dietary fiber intake (13.29 ± 8.89 g) compared to the MS group (8.79 ± 8.01 g), although this difference did not reach statistical significance (*p* = 0.066).

#### 3.4.3. Dietary Intake of Antioxidant Vitamins

**Figure 3:**
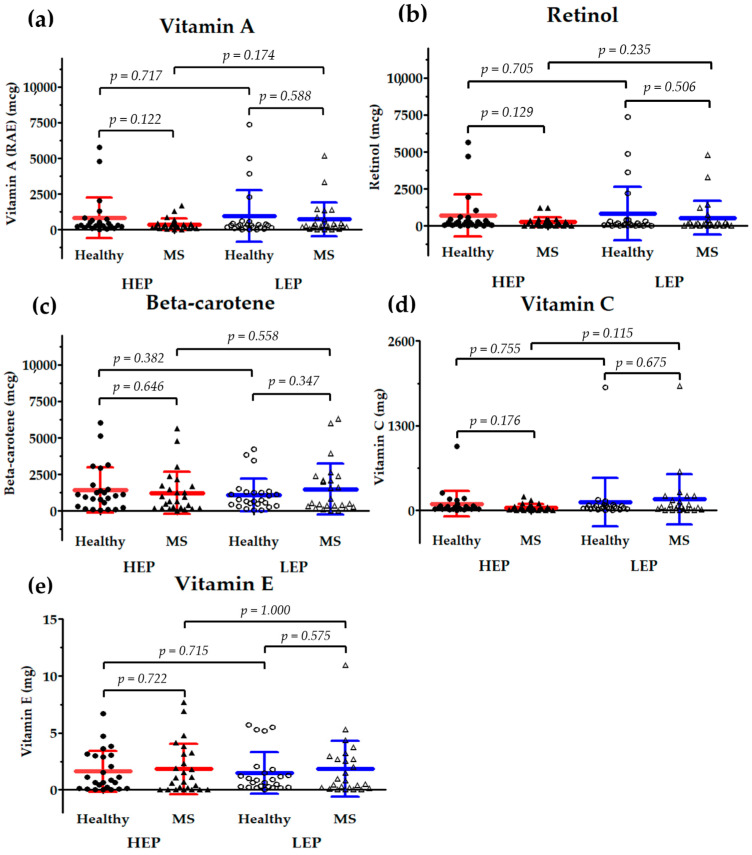
Scatter plots showing the levels of (**a**) vitamin A, (**b**) retinol, (**c**) β-carotene, (**d**) vitamin C, and (**e**) vitamin E daily intake in healthy subjects (*n* = 25) and individuals with metabolic syndrome (MS) (*n* = 25) during the high-PM2.5 exposure period (HEP) and low-PM2.5 exposure period (LEP). Data are presented as individual values with mean ± standard deviation (SD). Statistical comparisons between groups were performed, and *p*-values are indicated.

The results shown in Figure 3 are scatter plots that depict the levels of antioxidant vitamins, i.e., (a) vitamin A, (b) retinol, (c) β-carotene (provitamin A), (d) vitamin C, and (e) vitamin E, in healthy subjects and individuals with MS. These data were collected during the HEP and LEP. The statistical analysis reveals no significant differences in vitamin A, retinol, β-carotene, vitamin C, and vitamin E intakes between healthy subjects and those with MS under both exposure conditions.

However, during the HEP, the healthy group exhibited a trend toward a higher intake of vitamin A, retinol, and vitamin C compared to the MS group, but these differences were not statistically significant. Specifically, the intake of vitamin A in the healthy group was 831.67 ± 1410.24 µg, whereas in the MS group, it was 370.41 ± 395.12 µg (*p* = 0.122). Similarly, retinol intake was higher in the healthy group (697.89 ± 1419.57 µg) compared to the MS group (247.44 ± 326.18 µg), but this difference did not reach statistical significance (*p* = 0.129). The same trend was observed for vitamin C, where the healthy group had an intake of 99.56 ± 196.22 mg, while the MS group consumed 43.77 ± 52.25 mg, though this difference was also not statistically significant (*p* = 0.176).

Additionally, within the MS group, there was a tendency for the increased intake of vitamin A, retinol, and vitamin C during the LEP compared to the HEP, although these differences were not statistically significant. The intake of vitamin A increased from 370.41 ± 395.12 µg in the HEP to 721.19 ± 1172.24 µg in the LEP (*p* = 0.174), while retinol intake rose from 247.44 ± 326.18 µg in the HEP to 539.23 ± 1133.58 µg in the LEP (*p* = 0.235). Similarly, vitamin C intake in the MS group increased from 43.77 ± 52.25 mg in the HEP to 171.07 ± 384.80 mg in the LEP, though this change was not statistically significant (*p* = 0.115).

### 3.5. Effects of PM2.5 Exposure on Serum Inflammatory Markers

**Figure 4:**
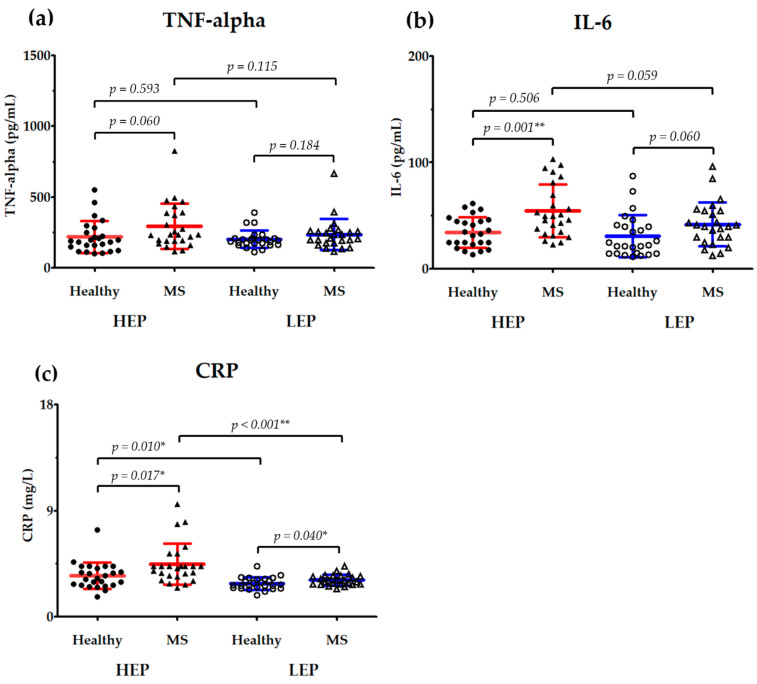
The serum levels of (**a**) tumor necrosis factor-alpha (TNF-α), (**b**) interleukin (IL)-6, and (**c**) C-reactive protein (CRP) in healthy subjects (*n* = 25) and individuals with metabolic syndrome (MS) (*n* = 25) during the high-PM2.5 exposure period (HEP) and low-PM2.5 exposure period (LEP). Data are presented as individual values with mean ± standard deviation (SD). Statistical comparisons between groups were performed, and *p*-values are indicated. * indicates a statistically significant difference with a *p*-value of <0.05, and ** indicates a statistically significant difference with a *p*-value of <0.01.

The effects of PM2.5 exposure on serum inflammatory markers, including (a) tumor necrosis factor-alpha (TNF-α), (b) interleukin (IL)-6, and (c) C-reactive protein (CRP), in healthy subjects and individuals with MS are illustrated in Figure 4. In order to evaluate the changes in systemic inflammation that are associated with air pollution exposure, the data were collected after the HEP and LEP.

When comparing between groups, TNF-α levels were higher in the MS group compared to the healthy group in both the HEP (293.57 ± 159.47 pg/mL vs. 218.26 ± 112.56 pg/mL, *p* = 0.060) and LEP (236.30 ± 109.32 pg/mL vs. 202.43 ± 61.82 pg/mL, *p* = 0.184). Although the differences were not statistically significant, this suggests a trend toward increased inflammation in individuals with MS. In the MS group, IL-6 levels were significantly higher than those of the healthy group during the HEP (54.47 ± 24.75 pg/mL vs. 34.49 ± 14.38 pg/mL, *p* = 0.001). In contrast, the difference in IL-6 levels in the LEP was no longer statistically significant (41.94 ± 20.54 pg/mL vs. 30.92 ± 19.87 pg/mL, *p* = 0.060). CRP levels were persistently higher in the MS group under both exposure conditions, as illustrated by the significant difference between the MS and healthy groups in both the HEP (4.49 ± 1.74 mg/L vs. 3.47 ± 1.11 mg/L, *p* = 0.017) and LEP (3.11 ± 0.45 mg/L vs. 2.82 ± 0.51 mg/L, *p* = 0.040).

The changes in inflammatory markers within groups under differing exposure conditions revealed that TNF-α levels did not significantly differ between the HEP and LEP in either the healthy group (218.26 ± 112.56 pg/mL vs. 202.43 ± 61.82 pg/mL, *p* = 0.593) or the MS group (293.57 ± 159.47 pg/mL vs. 236.30 ± 109.32 pg/mL, *p* = 0.115). In contrast to the HEP, the MS group exhibited a trend toward lower TNF-α levels during the LEP (*p* = 0.115). There was no significant difference in IL-6 levels (34.49 ± 14.38 pg/mL vs. 30.92 ± 19.87 pg/mL, *p* = 0.506) between the HEP and LEP in the healthy group. Conversely, the IL-6 levels in the MS group exhibited a decreasing trend in the LEP compared to in the HEP (54.47 ± 24.75 pg/mL vs. 41.94 ± 20.54 pg/mL, *p* = 0.059). In the healthy group, there was a significant decrease in CRP between the HEP and LEP (3.47 ± 1.11 mg/L vs. 2.82 ± 0.52 mg/L, *p* = 0.010). In the same vein, the CRP levels in the MS group were significantly lower in the LEP than in the HEP (4.49 ± 1.74 mg/L vs. 3.11 ± 0.45 mg/L, *p* < 0.001).

### 3.6. Correlation Between Nutrient Intake and Serum Inflammatory Markers

The Pearson correlation coefficients between dietary fiber, antioxidant vitamin intake (vitamin A, retinol, β-carotene, vitamin C, and vitamin E), and serum inflammatory markers (TNF-α, IL-6, and CRP) for healthy subjects and individuals with MS are presented in Table 5. In the healthy group, dietary fiber intake showed a significant negative correlation with IL-6 (*r* = −0.290, *p* < 0.05) and CRP (*r* = −0.308, *p* < 0.05), indicating that a higher fiber intake was associated with lower levels of inflammatory markers. Additionally, vitamin A intake exhibited a significant inverse correlation with IL-6 (*r* = −0.308, *p* < 0.05). Retinol intake was also significantly negatively correlated with IL-6 (*r* = −0.289, *p* < 0.05) but did not show significant associations with other inflammatory markers. Vitamin C intake demonstrated a significant negative correlation with CRP (*r* = −0.301, *p* < 0.05), suggesting that a higher vitamin C intake is linked to reduced systemic inflammation. Vitamin E intake showed a negative correlation with IL-6 (*r* = −0.235, *p* = 0.101), with a trend toward significance, and was significantly negatively correlated with CRP (*r* = −0.291, *p* < 0.05).

In the MS group, dietary fiber intake had a stronger negative correlation with CRP (*r* = −0.403, *p* < 0.01) and was significantly negatively correlated with IL-6 (*r* = −0.322, *p* < 0.05), reinforcing the association between a higher fiber intake and reduced inflammation, particularly in individuals with MS. However, vitamin A, retinol, β-carotene, vitamin C, and vitamin E intakes did not show significant correlations with TNF-α, IL-6, or CRP in the MS group. Despite this, negative trends were observed, particularly for β-carotene and vitamin C with IL-6 and CRP, suggesting a potential role of these nutrients in modulating inflammation, though these relationships did not reach statistical significance.

## 4. Discussion

PM2.5 poses significant health risks due to its ability to penetrate deep into the respiratory tract and enter the bloodstream, leading to various adverse health outcomes [1,2,3]. In Thailand, particularly in urban areas like Bangkok and northern regions such as Chiang Mai, PM2.5 levels often exceed the recommended safety thresholds, especially during the dry season when agricultural burning and forest fires are prevalent [28,29]. The World Health Organization (WHO) updated its air quality guidelines in 2021, recommending that annual mean PM2.5 concentrations should not exceed 5 μg/m^3^ and 24 h mean PM2.5 concentrations should not exceed 15 μg/m^3^ [30]. In response, Thailand revised its national standards in 2022 and 2023 to align with the WHO’s interim target-3 (IT-3), setting the 24 h PM2.5 standard at 37.5 μg/m^3^ and the annual mean at 15 μg/m^3^ [29]. Despite these regulatory efforts, actual PM2.5 concentrations frequently surpass these limits.

In this study, participants residing in Samoeng District, Chiang Mai Province, Thailand, were monitored during the HEP and LEP. When data were averaged across all five subdistricts, the HEP exhibited significantly higher mean PM2.5 concentrations and AQHI values compared to the LEP. During the HEP, the mean PM2.5 concentration was 50.86 ± 37.98 μg/m^3^, decreasing to 7.48 ± 6.91 μg/m^3^ in the LEP. Similarly, the mean AQHI dropped from 114.30 ± 50.93 in the HEP to 28.40 ± 22.53 in the LEP. These elevated levels exceed both WHO guidelines and Thailand’s national standards [29,30]. Such heightened PM2.5 levels are consistent with previous studies conducted in Northern Thailand during the dry season. Elevated PM2.5 levels have been associated with increased mortality rates from non-communicable diseases (NCDs) such as heart disease, hypertension, chronic lung disease, stroke, and diabetes [31,32,33]. A study examining Northern Thailand from 2017 to 2021 found a significant positive correlation between PM2.5 concentrations and mortality rates from these conditions. Notably, in Lampang, a correlation coefficient of 0.97 (95% CI = [0.66–0.99], *p* = 0.0048) was observed between PM2.5 levels and heart disease mortality rates, indicating a strong association. Similarly, Phayao exhibited a correlation coefficient of 0.9 (95% CI = [0.09–0.99], *p* = 0.0374) between PM2.5 concentrations and heart disease mortality rates [33].

MS, characterized by conditions such as central obesity, hypertension, elevated FBG, and dyslipidemia, increases susceptibility to the adverse effects of PM2.5 exposure [8]. Chronic exposure to PM2.5 has been linked to the development and exacerbation of MS components, with oxidative stress and systemic inflammation induced by PM2.5 playing significant roles in this process [9,10,11]. Although our study did not observe significant changes in metabolic parameters between the HEP and LEP in both healthy individuals and those with MS, a trend toward decreased systolic and diastolic blood pressure was noted in the healthy group when comparing the HEP to the LEP. This observation aligns with other studies indicating that PM2.5 exposure can elevate blood pressure, thereby increasing cardiovascular risk [34,35,36,37]. Regarding liver and kidney function markers, the observed increases in ALT and creatinine levels during the LEP remained within the normal reference ranges. The normal range of serum ALT levels was established as ≤40 IU/L [38], and in one study, serum creatinine levels ranged from 0.6 to 1.2 mg/dL for males and 0.5 to 1.1 mg/dL for females [39]. The increases observed in the present study may be attributed to factors beyond PM2.5 exposure, such as dietary changes, variations in physical activity, medication use, or underlying infections [40,41,42,43]. Further investigation is warranted to elucidate these potential contributing factors.

To assess the impact of PM2.5 exposure on systemic inflammation and confirm that observed effects are directly attributable to PM2.5 exposure, previous studies have utilized urinary biomarkers such as 1-hydroxypyrene (1-OHP), malondialdehyde (MDA), and 8-epi-prostaglandin F2α (8-epi-PGF2α) [44]. These biomarkers are widely used to monitor exposure and evaluate oxidative stress related to air pollution. In this study, these biomarkers were found to be significantly elevated during the HEP compared to the LEP. MDA and 8-epi-PGF2α are well-established indicators of oxidative stress induced by air pollution [45,46], whereas 1-OHP is a recognized biomarker for assessing internal exposure to polycyclic aromatic hydrocarbons (PAHs) [47]. The significant increase in these biomarkers during the HEP strongly indicates that air pollution had a direct impact on oxidative stress and systemic inflammation in exposed individuals.

The mechanisms underlying PM2.5-induced inflammation involve the activation of multiple oxidative stress-related signaling pathways [5,6,48]. Upon inhalation, PM2.5 generates ROS, leading to oxidative damage that triggers the activation of transcription factors such as NF-κB. This activation results in the production of pro-inflammatory cytokines, including TNF-α and IL-6 [5,6,48]. The elevated levels of these cytokines subsequently stimulate the liver to produce acute-phase proteins such as CRP, which serves as a key biomarker of systemic inflammation [7]. A study investigating the effects of PM2.5 exposure found significant positive associations between particulate matter and increased levels of CRP, IL-6, and soluble TNF receptor-2 (sTNFR-2), highlighting the inflammatory responses elicited by PM2.5 [49].

The findings of this study are consistent with the existing literature demonstrating that PM2.5 exposure leads to systemic inflammation. During the HEP, both healthy individuals and those with MS exhibited significantly higher CRP levels compared to those during the LEP. These results support previous studies that have reported associations between short-term PM2.5 exposure and elevated inflammatory markers [50,51,52]. For example, research has demonstrated that increased short-term exposure to air pollution correlates with higher CRP levels, indicating an acute inflammatory response to PM2.5 [51].

Exposure to PM2.5 initiates systemic inflammation primarily through the elevated production of IL-6. Upon PM2.5 exposure, IL-6 binds to its specific receptor complex, IL-6 receptor (IL-6R) and glycoprotein 130 (gp130), subsequently activating two major signaling pathways involved in inflammation: the JAK1/STAT3 pathway in endothelial cells and the STAT3/SOCS3 pathway in hepatic cells [53,54].

In endothelial cells, PM2.5 activates the IL-6-dependent JAK1/STAT3 signaling pathway, resulting in increased phosphorylation and the activation of signal transducer and activator of transcription 3 (STAT3). The activated STAT3 promotes the expression of critical adhesion molecules and procoagulant factors such as intercellular adhesion molecule-1 (ICAM-1), vascular cell adhesion molecule-1 (VCAM-1), and tissue factor (TF). This molecular cascade contributes to endothelial activation, enhancing leukocyte adherence to the vascular endothelium, thereby leading to vascular inflammation and dysfunction, a critical step in the pathogenesis of CVD linked to PM2.5 exposure [53].

In hepatic cells, PM2.5 exposure induces a systemic elevation in IL-6 levels, subsequently triggering the IL-6-mediated STAT3/SOCS3 signaling pathway. Specifically, IL-6 activates STAT3, leading to the increased expression of suppressor of cytokine signaling 3 (SOCS3) in liver tissues. SOCS3 interferes with insulin signaling by impairing insulin receptor substrate-1 (IRS-1) phosphorylation. Consequently, insulin resistance is aggravated, reducing glucose uptake and glycogen synthesis in hepatocytes, primarily through the downregulation of GLUT2 and GLUT4 glucose transporters. Additionally, hepatic oxidative stress and macrophage infiltration are increased, further exacerbating metabolic dysfunction associated with type 2 diabetes mellitus [54].

Although no significant differences in IL-6 levels between the HEP and LEP were observed in healthy individuals within our study, IL-6 levels in the MS group showed an increasing trend during the HEP (*p* = 0.059), suggesting heightened inflammatory responses in metabolically compromised individuals. Furthermore, reduced PM2.5 exposure appeared to attenuate inflammation, as indicated by the observed downward trend in IL-6 levels during the LEP. These findings collectively highlight the crucial role of IL-6, which appears to mediate inflammatory responses through both the JAK1/STAT3 and STAT3/SOCS3 signaling pathways. This helps clarify the complex interplay between PM2.5 exposure and inflammatory processes that potentially contribute to cardiovascular and metabolic diseases. Further investigations into these molecular mechanisms are essential and should be pursued in future studies.

Recurrent PM2.5 pollution in Northern Thailand poses a significant public health concern. Despite government efforts to mitigate this issue by reducing agricultural burning, persistent wildfires continue to deteriorate air quality, leading to unavoidable exposure to PM2.5 [28,29]. It is well established that PM2.5 exposure induces systemic inflammation and oxidative stress, both of which contribute to the development of chronic diseases [9,10,11,33]. However, research on nutritional interventions, such as antioxidant-rich nutrient intake, as a potential strategy to counteract the inflammatory effects of PM2.5 exposure remains limited, warranting further investigation [17,18,19].

In our study, we observed no significant differences in the intakes of antioxidant vitamins—specifically, vitamin A, retinol, β-carotene, vitamin C, and vitamin E—between healthy individuals and those with MS during the HEP and LEP. However, among healthy participants, an inverse relationship was identified between the intakes of vitamin A and retinol and levels of IL-6, as well as between the intakes of vitamin C and vitamin E and levels of CRP. This suggests that the higher consumption of these antioxidant vitamins may be associated with reduced systemic inflammation in healthy individuals. Conversely, no such associations were observed in participants with MS, indicating a potential differential response to antioxidant intake based on metabolic health status.

Another concern arising from this study is the excessive sodium intake observed among both healthy individuals and those with MS residing in Samoeng District. The average sodium consumption exceeded 3000 mg/day, surpassing the recommended limit of 2000 mg/day [55]. High sodium intake is well documented as a contributing factor to hypertension, a major risk factor for CVDs [56]. Regarding inflammation, the association between high sodium intake and inflammatory markers remains inconclusive. Some studies suggest that excessive sodium consumption may exacerbate inflammation [57,58], while others have found no significant correlation [59,60]. Given the well-established relationship between high sodium intake and hypertension [56], it is crucial to implement dietary interventions aimed at reducing sodium consumption among the population of Samoeng District. Strategies such as promoting the consumption of fresh, unprocessed foods and minimizing processed food intake could effectively lower sodium consumption and, consequently, reduce the risk of hypertension and related health complications.

Our findings also revealed that participants with MS had a higher dietary fiber intake during the LEP compared to during the HEP. Moreover, dietary fiber intake was inversely correlated with the levels of IL-6 and CRP in healthy individuals and showed a strong inverse correlation with CRP in those with MS (*r* = −0.403, *p* < 0.01). This suggests that the reduction in inflammatory markers is not only due to the decrease in PM2.5 levels but also influenced by an increased dietary fiber intake, which may have contributed to the observed reduction in inflammation. This is evidenced by the significant decrease in CRP levels (*p* < 0.001) and the downward trends in IL-6 (*p* = 0.059) and TNF-α (*p* = 0.115) among individuals with MS during the LEP compared to the HEP. These observations align with those of previous studies that demonstrate that increased dietary fiber consumption is associated with lower concentrations of inflammatory markers [61,62,63]. For instance, a study involving 4125 older adults found that higher total fiber and cereal fiber intakes were linked to reduced inflammation, as evidenced by the lower levels of CRP, IL-6, and tumor necrosis factor-alpha receptor-2 (TNF-α-R2) [63].

An increased dietary fiber intake often corresponds to a higher consumption of fruits and vegetables, which are rich in phytochemicals such as phenolic compounds, flavonoids, and anthocyanins [64]. These compounds possess potent antioxidant and anti-inflammatory properties [65]. For example, protocatechuic acid, a phenolic compound found in various plants, has demonstrated antioxidant and anti-inflammatory activities, including the inhibition of chemically induced liver toxicity in vivo [66]. Furthermore, phytochemicals have been reported to mitigate PM2.5-induced adverse effects through various molecular mechanisms, including antioxidant and anti-inflammatory pathways [67]. A review highlighted that compounds such as polyphenols and carotenoids can counteract oxidative stress and suppress pro-inflammatory responses induced by PM2.5 exposure [67]. Additionally, curcumin, a bioactive compound present in turmeric, has been found to protect human bronchial epithelial cells from PM2.5-induced oxidative damage and inflammation by activating the nuclear factor erythroid 2-related factor 2 (NRF2) pathway [68].

This study has several important limitations that should be addressed in future research. Firstly, the lack of detailed chemical characterization of PM2.5 and corresponding biomarkers in participants’ blood limits our ability to confirm specific associations between particular PM2.5 constituents and the observed health effects. Nevertheless, the measurement of biomarkers such as urinary 1-OHP, MDA, and 8-epi-PGF2α provided supportive evidence linking PM2.5 exposure to oxidative stress and inflammation [44]. Secondly, this study utilized only three inflammatory markers (IL-6, TNF-α, and CRP), with significant results observed primarily for CRP, possibly due to its involvement in the systemic inflammatory response. Future research should incorporate a wider array of inflammatory, oxidative stress, and endothelial dysfunction biomarkers to better elucidate underlying mechanisms. Thirdly, the relatively small sample size may have increased variability and reduced the generalizability of the findings. Subsequent studies should therefore aim to include larger and more representative populations, along with comprehensive chemical and biological analyses, to robustly investigate the health impacts and mechanistic pathways associated with PM2.5 exposure. Additionally, confounding factors, such as physical activity, may have influenced our results. Individuals with higher physical activity levels might possess greater resilience against inflammation and oxidative stress, potentially affecting the observed outcomes. However, physical activity performed outdoors during the HEP could paradoxically increase exposure. Moreover, occupational exposure to air pollutants should also be considered, as individuals working outdoors or in occupations involving frequent exposure to dust (e.g., street sweepers) may experience higher PM2.5 exposure levels compared to those working indoors. These confounding factors were not assessed in this study, and future research should systematically collect data on these variables to enhance the accuracy and reliability of findings.

Collectively, these findings underscore the potential of dietary modifications, particularly increasing the intakes of dietary fiber and antioxidant-rich foods, as effective strategies to attenuate inflammation and oxidative stress associated with PM2.5 exposure. Such interventions could be especially beneficial for individuals with metabolic disorders, offering a practical approach to mitigate the health risks posed by air pollution in Northern Thailand. Future research should further explore these associations and consider longitudinal studies to establish causality and inform public health recommendations.

## 5. Conclusions

This study highlights the impact of PM2.5 exposure and dietary factors on systemic inflammation, particularly in individuals with MS. The study findings indicate that individuals with MS exhibit heightened inflammatory responses, as reflected by the elevated CRP and IL-6 levels, with CRP showing significant reductions during the low-PM2.5 exposure period. Notably, dietary fiber intake demonstrated a strong inverse relationship with systemic inflammation, particularly in the MS group, whereas antioxidant vitamins showed a protective effect only in healthy individuals. These results suggest that dietary fiber plays a crucial role in mitigating inflammation associated with PM2.5 exposure, especially in metabolically compromised individuals. Given the increasing burden of air pollution, promoting fiber-rich diets may serve as an effective nutritional strategy to counteract the inflammatory effects of PM2.5, thereby improving metabolic health and reducing the risk of associated chronic diseases. Further research is warranted to explore the long-term benefits of dietary interventions in populations exposed to high levels of air pollution.

## Figures and Tables

**Table 1:** Demographic characteristics, lifestyle factors, and health conditions of subjects.

Variables	Healthy (*n* = 25)	Metabolic Syndrome (*n* = 25)	Total (*n* = 50)	*p*
Gender, N (%)				0.225
Male	10 (40.0)	6 (24.0)	16 (32.0)	
Female	15 (60.0)	19 (76.0)	34 (68.0)	
Age (years), mean ± SD	48.3 ± 14.3	52.2 ± 8.3	50.72 ± 11.8	0.177
Alcohol consumption, N (%)				0.140
Never	6 (24.0)	12 (48.0)	18 (36.0)	
Yes	19 (76.0)	13 (52.0)	32 (64.0)	
Smoking, N (%)				0.411
Never smoked	18 (72.0)	20 (80.0)	38 (76.0)	
Smoker	4 (16.0)	1 (4.0)	5 (10.0)	
Former smoker	4 (12.0)	4 (16.0)	7 (14.0)	
Underlying diseases, N (%)				
Diabetes	0	5 (10.0)	5 (10.0)	0.050
Hypertension	2 (8.0)	14 (56.0)	16 (32.0)	0.001 *
Dyslipidemia	1 (4.0)	7 (28.0)	8 (16.0)	0.049 *
Other diseases (gastroesophageal reflux disease, allergy/asthma, herniated disk, thyroid disorder)	6 (24.0)	2 (8.0)	8 (16.0)	0.446

* Shows a significant difference between healthy subjects and subjects with metabolic syndrome with a *p*-value of <0.05.

**Table 2:** Physical and biochemical parameters in healthy subjects and individuals with MS after high- and low-PM2.5 exposure periods.

Variables	HEP			LEP			*p* ^3^	*p* ^4^
	Healthy (Mean ± SD, *n* = 25)	MS (Mean ± SD, *n* = 25)	*p* ^1^	Healthy (Mean ± SD, *n* = 25)	MS (Mean ± SD, *n* = 25)	*p* ^2^		
Body height (cm)	156.2 ± 7.6	156.6 ± 7.4	0.923	157.9 ± 7.6	156.2 ± 6.5	0.313	0.390	0.758
Body weight (kg)	59.4 ± 14.4	71.5 ± 16.3	0.001 **	60.0 ± 10.3	75.8 ± 22.3	0.004 **	0.555	0.640
Body mass index, BMI (kg/m^2^)	24.3 ± 5.0	29.1 ± 5.7	<0.001 **	24.0 ± 3.4	30.7 ± 6.5	<0.001 **	0.747	0.400
Waist circumference, WC (cm)	79.9 ± 13.7	92.1 ± 12.5	<0.001 **	79.9 ± 8.9	96.7 ± 15.0	<0.001 **	0.575	0.255
Hip circumference, HC (cm)	93.6 ± 10.5	101.9 ± 9.6	<0.001 **	94.0 ± 7.6	105.0 ± 13.8	0.004 **	0.509	0.521
Systolic blood pressure, SBP (mmHg)	134.6 ± 19.8	137.9 ± 19.1	0.574	125.6 ± 19.6	130.9 ± 14.3	0.289	0.056	0.256
Diastolic blood pressure, DBP (mmHg)	83.8 ± 10.6	86.1 ± 14.2	0.377	78.2 ± 10.2	83.0 ± 8.2	0.116	0.057	0.245
Heart rate, HR (bpm)	74.0 ± 9.3	72.8 ± 9.6	0.534	71.0 ± 8.4	76.4 ± 0.2	0.065	0.249	0.228
Fasting blood glucose, FBG (mg/dL)	83.7 ± 7.2	114.7 ± 59.6	0.004 **	86.3 ± 11.7	106.5 ± 18.2	<0.001 **	0.612	0.245
Total cholesterol, TC (mg/dL)	206.8 ± 29.8	193.0 ± 30.7	0.103	206.9 ± 40.8	209.2 ± 34.2	0.934	0.921	0.240
Triglyceride, TG (mg/dL)	133.0 ± 78.5	191.4 ± 95.1	0.003 **	126.1 ± 42.7	197.8 ± 73.8	<0.001 **	0.640	0.438
High-density lipoprotein cholesterol, HDL-C (mg/dL)	55.2 ± 11.1	44.9 ± 10.1	0.001 **	55.7 ± 13.0	48.6 ± 9.3	0.034 *	0.848	0.190
Low-density lipoprotein cholesterol, LDL-C (mg/dL)	122.7 ± 32.0	110.3 ± 28.5	0.118	113.1 ± 33.5	114.6 ± 28.0	0.967	0.283	0.728
Alanine aminotransferase, ALT (U/L)	27.7 ± 17.9	26.7 ± 12.4	0.560	28.5 ± 13.7	33.3 ± 14.2	0.090	0.330	0.040 *
Aspartate aminotransferase, AST (U/L)	34.4 ± 11.6	34.4 ± 15.4	0.466	33.8 ± 7.4	32.5 ± 10.6	0.248	0.612	0.883
Serum creatinine (mg/dL)	0.8 ± 0.2	0.8 ± 0.2	0.404	0.9 ± 0.2	0.8 ± 0.1	0.047 *	0.143	0.968
Blood urea nitrogen, BUN (mg/dL)	13.5 ± 3.6	13.5 ± 3.5	0.793	14.5 ± 3.5	13.6 ± 3.1	0.400	0.217	0.852

^1^ *p*-value represents the statistical comparison between the healthy and MS groups following the high-PM2.5 exposure period (HEP). ^2^ *p*-value represents the statistical comparison between the healthy and MS groups following the low-PM2.5 exposure period (LEP). ^3^ *p*-value represents the statistical comparison between the HEP and LEP in the healthy group. ^4^ *p*-value represents the statistical comparison between the HEP and LEP in the MS group. * indicates a statistically significant difference with a *p*-value of <0.05, and ** indicates a statistically significant difference with a *p*-value of <0.01.

**Table 3:** Distribution of energy intake from macronutrients in healthy subjects and individuals with MS during high- and low-PM2.5 exposure periods.

Macronutrients	HEP			LEP			*p* ^3^	*p* ^4^
	Healthy (Mean ± SD, *n* = 25)	MS (Mean ± SD, *n* = 25)	*p* ^1^	Healthy (Mean ± SD, *n* = 25)	MS (Mean ± SD, *n* = 25)	*p* ^2^		
Carbohydrate (%)	56.96 ± 9.23	54.24 ± 17.34	0.493	53.48 ± 16.82	53.25 ± 13.94	0.960	0.357	0.745
Protein (%)	17.44 ± 5.13	19.20 ± 11.62	0.491	18.69 ± 6.10	19.68 ± 7.57	0.613	0.477	0.786
Fat (%)	25.60 ± 8.26	26.55 ± 11.70	0.741	27.83 ± 12.74	27.06 ± 9.23	0.808	0.464	0.857

^1^ *p*-value represents the statistical comparison between the healthy and MS groups during the high-PM2.5 exposure period (HEP). ^2^ *p*-value represents the statistical comparison between the healthy and MS groups during the low-PM2.5 exposure period (LEP). ^3^ *p*-value represents the statistical comparison between the HEP and LEP in the healthy group. ^4^ *p*-value represents the statistical comparison between the HEP and LEP in the MS group.

**Table 4:** Average daily energy and nutrient intake in healthy subjects and individuals with MS during high- and low-PM2.5 exposure periods.

Variables	HEP			LEP			*p* ^3^	*p* ^4^
	Healthy (Mean ± SD, *n* = 25)	MS (Mean ± SD, *n* = 25)	*p* ^1^	Healthy (Mean ± SD, *n* = 25)	MS (Mean ± SD, *n* = 25)	*p* ^2^		
Energy (kcal)	1704.77 ± 660.21	1550.64 ± 684.87	0.422	1629.87 ± 548.25	1567.72 ± 535.05	0.687	0.625	0.909
Carbohydrates (g)	242.50 ± 103.51	214.92 ± 131.01	0.413	218.18 ± 109.54	209.37 ± 86.08	0.753	0.422	0.847
Sugars (g)	37.28 ± 28.55	22.88 ± 25.60	0.067	31.83 ± 24.20	35.30 ± 38.01	0.702	0.390	0.155
Proteins (g)	72.04 ± 29.73	67.42 ± 27.93	0.573	74.84 ± 31.60	74.46 ± 33.10	0.967	0.725	0.355
Fats (g)	49.62 ± 27.69	46.81 ± 29.52	0.730	50.87 ± 31.73	48.04 ± 26.44	0.734	0.875	0.862
Total saturated fatty acids (g)	12.23 ± 11.38	10.20 ± 8.91	0.486	13.40 ± 10.83	12.65 ± 10.35	0.803	0.721	0.416
Cholesterol (mg)	346.22 ± 275.30	321.30 ± 305.16	0.763	357.70 ± 315.13	281.42 ± 221.45	0.327	0.885	0.538
Calcium (mg)	503.48 ± 431.13	415.59 ± 240.97	0.378	403.41 ± 337.95	458.10 ± 453.31	0.631	0.366	0.686
Phosphorus (mg)	848.08 ± 313.46	752.47 ± 344.61	0.310	855.26 ± 409.06	893.73 ± 416.64	0.743	0.945	0.123
Iron (mg)	12.32 ± 5.33	13.36 ± 6.60	0.543	13.35 ± 6.94	14.66 ± 5.77	0.470	0.514	0.389
Potassium (mg)	1711.90 ± 747.67	1370.82 ± 764.01	0.117	1620.98 ± 746.08	1805.51 ± 1190.02	0.514	0.609	0.108
Sodium (mg)	3417.59 ± 1635.23	3307.41 ± 1196.97	0.787	3494.50 ± 1922.22	3546.37 ± 1359.04	0.913	0.872	0.434
Magnesium (mg)	58.65 ± 47.18	45.96 ± 49.92	0.360	52.34 ± 47.09	71.57 ± 66.48	0.244	0.662	0.125
Copper (mg)	0.86 ± 0.48	0.67 ± 0.36	0.126	0.98 ± 1.06	0.94 ± 0.78	0.879	0.574	0.142
Selenium (μg)	49.91 ± 45.23	42.10 ± 47.12	0.553	53.49 ± 47.18	45.99 ± 52.42	0.597	0.760	0.698
Zinc (mg)	5.09 ± 2.00	4.68 ± 2.10	0.479	4.99 ± 2.79	4.87 ± 2.39	0.869	0.856	0.724
Vitamin B1 (mg)	1.34 ± 0.89	1.72 ± 3.75	0.627	1.30 ± 1.05	3.92 ± 12.87	0.315	0.880	0.420
Vitamin B2 (mg)	1.25 ± 0.67	0.98 ± 0.39	0.086	1.14 ± 0.57	1.29 ± 1.02	0.530	0.533	0.142
Vitamin B6 (mg)	0.75 ± 1.11	0.40 ± 0.32	0.137	0.78 ± 0.83	0.74 ± 1.08	0.883	0.897	0.098
Vitamin B12 (mg)	2.27 ± 3.17	1.43 ± 1.54	0.241	3.55 ± 6.62	1.75 ± 1.76	0.195	0.361	0.387
Niacin (mg)	17.46 ± 10.82	13.09 ± 7.96	0.110	15.32 ± 8.30	16.08 ± 12.57	0.803	0.363	0.298
Dietary fiber (g)	13.29 ± 8.89	8.79 ± 8.01	0.066	11.73 ± 9.63	14.05 ± 12.56	0.468	0.521	0.037 *

^1^ *p*-value represents the statistical comparison between the healthy and MS groups during the high-PM2.5 exposure period (HEP). ^2^ *p*-value represents the statistical comparison between the healthy and MS groups during the low-PM2.5 exposure period (LEP). ^3^ *p*-value represents the statistical comparison between the HEP and LEP in the healthy group. ^4^ *p*-value represents the statistical comparison between the HEP and LEP in the MS group. * indicates a statistically significant difference with a *p*-value of <0.05.

**Table 5:** Pearson correlation coefficients (*r*) between dietary fiber, antioxidant vitamins, and serum inflammatory markers.

Groups	Nutrient Intake	Serum Inflammatory Markers		
		TNF-α	IL-6	CRP
Healthy	Dietary fiber	−0.242	−0.290 *	−0.308 *
		0.090	0.041	0.029
	Vitamin A	−0.052	−0.308 *	−0.117
		0.720	0.030	0.417
	Retinol	−0.029	−0.289 *	−0.106
		0.843	0.042	0.465
	β-carotene	−0.237	−0.248	−0.098
		0.098	0.082	0.497
	Vitamin C	−0.076	−0.223	−0.301 *
		0.600	0.120	0.033
	Vitamin E	−0.153	−0.235	−0.291 *
		0.288	0.101	0.040
MS	Dietary fiber	−0.182	−0.322 *	−0.403 **
		0.206	0.022	0.004
	Vitamin A	−0.173	−0.160	−0.270
		0.230	0.267	0.058
	Retinol	−0.128	−0.121	−0.217
		0.375	0.404	0.130
	β-carotene	−0.183	−0.244	−0.244
		0.203	0.088	0.087
	Vitamin C	−0.111	−0.226	−0.202
		0.443	0.114	0.159
	Vitamin E	−0.090	−0.108	−0.231
		0.535	0.457	0.107

** Correlation is significant at the 0.01 level (two-tailed). * Correlation is significant at the 0.05 level (two-tailed).

## Data Availability

The data presented in this study are available upon request from the corresponding author.

## Abbreviations

The following abbreviations are used in this manuscript:

8-epi-PGF2α	8-epi-prostaglandin F2α
NTAQHI	Northern Thailand Air Quality Health Index
SREBP1	sterol regulatory element-binding protein 1
ELISA	enzyme-linked immunosorbent assay
RIHES	Research Institute for Health Sciences
PPARα	peroxisome proliferator-activated receptor alpha
PPARγ	peroxisome proliferator-activated receptor gamma
SOCS3	suppressor of cytokine signaling 3
STAT3	signal transducer and activator of transcription 3
ICAM-1	intercellular adhesion molecule-1
VCAM-1	vascular cell adhesion molecule-1
AQHI	Air Quality Health Index
CVDs	cardiovascular diseases
GLUT	glucose transporter
NCDs	non-communicable diseases
PAHs	polycyclic aromatic hydrocarbons
HDL-C	high-density lipoprotein cholesterol
LDL-C	low-density lipoprotein cholesterol
TNF-α	tumor necrosis factor-alpha
JAK1	Janus kinase 1
1-OHP	1-hydroxypyrene
ALT	alanine aminotransferase
AQI	Air Quality Index
AST	aspartate aminotransferase
BMI	body mass index
BUN	blood urea nitrogen
CRP	C-reactive protein
DBP	diastolic blood pressure
FBG	fasting blood glucose
HEP	high-PM2.5 exposure period
LEP	low-PM2.5 exposure period
MDA	malondialdehyde
ROS	reactive oxygen species
SBP	systolic blood pressure
VAT	visceral adipose tissue
WHO	World Health Organization
IL-6	interleukin-6
NF-κB	nuclear factor-kappa B
PM2.5	fine particulate matter
Cu	copper
HC	hip circumference
HR	heart rate
MS	metabolic syndrome
SD	standard deviation
TC	total cholesterol
TG	triglyceride
WC	waist circumference
Zn	zinc
